# MYC-activated RNA N6-methyladenosine reader IGF2BP3 promotes cell proliferation and metastasis in nasopharyngeal carcinoma

**DOI:** 10.1038/s41420-022-00844-6

**Published:** 2022-02-08

**Authors:** Mingyu Du, Yi Peng, Yang Li, Wenyue Sun, Huanfeng Zhu, Jing Wu, Dan Zong, Lirong Wu, Xia He

**Affiliations:** The Affiliated Cancer Hospital of Nanjing Medical University, Jiangsu Cancer Hospital, Jiangsu Institute of Cancer Research, 42 Baiziting Road, Nanjing, Jiangsu China

**Keywords:** Tumour biomarkers, Head and neck cancer

## Abstract

N6-Methyladenosine (m6A) modification is the most abundant RNA modification in eukaryotic cells. IGF2BP3, a well-known m6A reader, is deregulated in many cancers, but its role in nasopharyngeal carcinoma (NPC) remains unclear. In this work, IGF2BP3 was upregulated in NPC tissues and cells. The high level of IGF2BP3 was positively related to late clinical stages, node metastasis, and poor outcomes. Moreover, IGF2BP3 accelerated NPC cell tumor progression and metastasis in vitro and vivo. Upstream mechanism analyses indicated that the high expression of IGF2BP3 in head and neck tumors was mainly due to mRNA level amplification. Luciferase assay and chromatin immunoprecipitation assay (CHIP) depicted that MYC was effectively bound to the promoter of IGF2BP3, thereby improving its transcriptional activity. Results also showed that IGF2BP3 was not only positively correlated with KPNA2 expression but also modulated the expression of KPNA2. m6A RNA immunoprecipitation (MeRIP) and RNA stability experiments verified that silencing IGF2BP3 significantly inhibited the m6A modification level of KPNA2, thereby stabilizing the mRNA stability of KPNA2. Rescue experiments proved that the effect of inhibiting or overexpressing IGF2BP3 on NPC cells was partly reversed by KPNA2. Collectively, MYC-activated IGF2BP3 promoted NPC cell proliferation and metastasis by influencing the stability of m6A-modified KPNA2. Our findings offer new insights that IGF2BP3 may serve as a new molecular marker and potential therapeutic target for NPC treatment.

## Introduction

Nasopharyngeal carcinoma (NPC) is a prevalent and malignant head-and-neck tumor arising from the epithelial lining of nasopharynx. Approximately 133,354 cases of NPC were diagnosed, and 80,008 people died from the disease in 2020 [1]. Globally, NPC exhibits distinctive ethnic and geographical differences, with a particularly high incidence in North Africa, Southeast Asia, and southern China [2, 3]. The occurrence and development of NPC are often triggered by the interaction between genetic polymorphisms and multiple environmental risk factors, especially Epstein–Barr virus (EBV) infection [4, 5]. Despite the advances in integrative therapies, including radiotherapy, chemotherapy, and targeted therapy that improve the 5-year overall survival rate of patients with NPC to 90%, the prognosis of patients with distant metastasis remains poor [6]. Therefore, the molecular mechanism underlying the metastasis of NPC should be elucidated for precise treatment.

N^6^-Methyladenosine (m^6^A), methylated at the N^6^position of adenosine, is the most frequent post-transcriptional modification in mRNA [7]. m6A mainly occurs in the conserved sequence of the RGACH motif (R = G/A; H = A/C/U) [8] and participates in multiple translation regulations. The regulators of m6A can be catalyzed into the following types: methyltransferase complex (writers), demethylases (erasers), and m6A-binding proteins (readers) [9]. m6A methyltransferase is a complex that contains multiple subunits, where METTL3 is the only catalytic subunit. RNA m6A modification can be removed by FTO and ALKBH5. m6A readers, such as YTH domain family proteins, IGF2BPs, HNRNPC, HNRNPA2/B1, and eIF3, are needed to perform biological functions. These regulators lead to the dynamic and reversible modification process of m6A. The dysregulation of these m6A modulators plays a dual role as carcinogen or tumor suppressor in various types of tumors. For instance, METTL3 decreases the expression of PHLPP2 (a negative AKT regulator) and increases the expression of mTORC2 (a positive AKT regulator) to stimulate AKT activation and suppress tumorigenesis and invasion in endometrial cancer [10]. METTL3 also regulates its downstream target SOCS2 and triggers hepatocellular carcinoma cell proliferation and migration via an m6A-YTHDF2-dependent mechanism [11]. Another study revealed that m6A regulators perform important roles in the progression of several cancer types. M6A-associated proteins exhibit widespread epigenetic alterations across 33 cancer types, in which the m6A reader IGF2BP3 (also known as IMP3, KOC) is significantly correlated with adverse outcomes [12]. Although increasing lines of evidence have proved that the deviant expression of m6A modification may be the cause of tumor progression [13–15], the mechanism through which m6A regulators, especially IGF2BP3, function in NPC tumorigenesis remains unknown.

In this research, we investigated the basic expression and biological functions of IGF2BP3 in NPC. We identified for the first time an oncogenic axis consisting of MYC-IGF2BP3-KPNA2. Finally, the clinical and therapeutic significance of these putative prognostic biomarkers was elaborated. We also explored the molecular mechanism by which IGF2BP3 regulates target mRNA transcripts in an m6A-dependent manner. This work offers a potential strategy to obtain a better prognosis for patients with NPC.

## Results

### IGF2BP3 is overexpressed in NPC and associated with poor prognosis

**Fig. 1:**
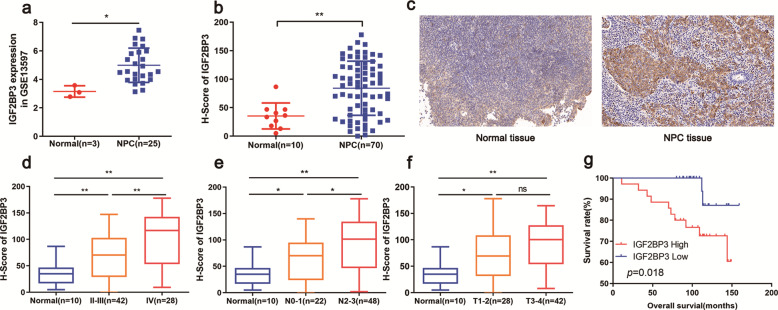
Identification of an m6A reader IGF2BP3 whose overexpression is associated with NPC prognosis. **a** Bioinformation analysis showed the IGF2BP3 expression in normal nasopharyngeal tissues (*n* = 3) and the NPC tissues (*n* = 25) based on the GEO datasets (GSE13597). **b**, **c** The expression level of IGF2BP3 was detected by IHC analysis (**c**) and assessed by H-score (**b**) in normal tissues (*n* = 10) and NPC tissues (*n* = 70). **d**–**f** IGF2BP3 expression was analyzed in normal and NPC patients regarding tumor grade (**d**), N stage (**e**), and T stage (**f**), using H-score. **g** Kaplan–Meier analysis showed that the expression of IGF2BP3 was a predictive factor of overall survival in NPC. Data was shown as the mean ± standard deviation of three independent experiments. *P < 0.05, **P < 0.01.

To characterize the role of IGF2BP3 in NPC carcinogenesis, we screened data from the GEO datasets and our collected NPC tissues. As shown in GSE13597, the IGF2BP3 expression was significantly higher in NPC tissues than in the controls (Fig. 1a). Based on analysis of our collected tissues, the expression of IGF2BP3 in 70 NPC tissues was higher than that in the normal controls (Fig. 1b, c). Statistical assays indicated that the high level of IGF2BP3 was positively associated with advanced TNM stages and N classification (Fig. 1d, e). Meanwhile, IGF2BP3 expression showed no obvious links with age, sex, and T classification (Table 1, Fig. 1f). Kaplan–Meier survival assays revealed that higher IGF2BP3 expression indicates worse outcomes (Fig. 1g). Univariate and multivariate analyses identified that IGP2BP3 expression is an independent prognostic factor for patients with NPC (Table 2). Herein, we assumed that IGF2BP3 exerts irreplaceable roles in the progression of NPC.

**Table 1:** Correlation between IGF2BP3 expression and clinical characteristics of NPC patients.

Characteristics	No of patients (*n* = 70)	IGF2BP3low group	IGF2BP3high group	*P* value
Age(years)				
≤49	37	20	17	0.473
>49	33	15	18	
Sex				
Female	14	8	6	0.765
Male	56	27	29	
TNM stage				
II-III	42	27	15	0.003
IV	28	8	20	
T classification				
T1-T2	28	17	11	0.267
T3-T4	42	18	24	
N classification				
N0-N1	22	15	7	0.039
N2-N3	48	20	28

**Table 2:** Prognostic factors for univariate analysis and multivariate analysis.

Characteristics	No.	univariate analysis		multivariate analysis	
		HR(95%CI)	*P*	HR(95%CI)	*P*
Age(years)					
≤49	37	1		1	
>49	33	0.909(0.292–2.812)	0.865	0.801(0.214–2.994)	0.742
Sex					
Female	14	1		1	
Male	56	2.442(0.312–19.083)	0.395	2.884(0.363–22.896)	0.316
TMN stage					
II-III	42	1		1	
IV	28	1.179(0.374–3.723)	0.779	0.694(0.166–2.908)	0.617
T classification					
T1-2	28	1		1	
T3-4	42	1.024(0.324–3.037)	0.968	0.825(0.191–3.559)	0.796
N classification					
N0-1	22	1		1	
N2-3	48	5.474(0.706–42.421)	0.104	4.962(0.619–39.790)	0.132
IGF2BP3 expression					
Low	37	1		1	
High	35	5.178(1.132–23.682)	0.034	5.423(1.064–27.637)	0.042

### Knockdown of IGF2BP3 represses NPC migration and invasion in vitro

**Fig. 2:**
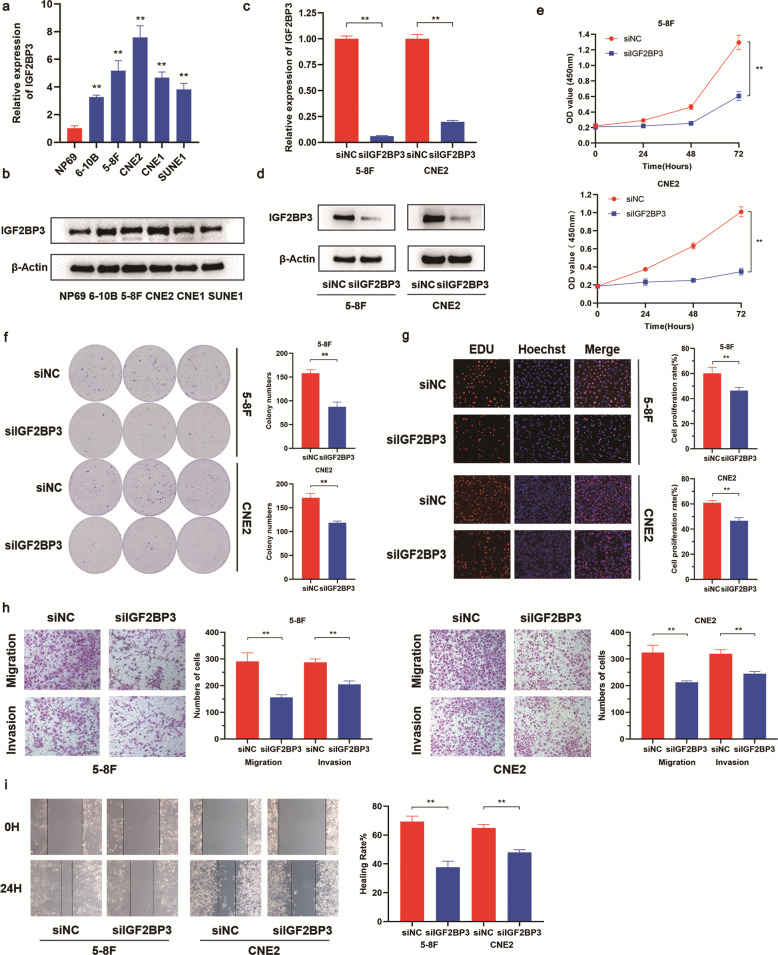
IGF2BP3 is upregulated in NPC cells and promotes proliferation and metastasis. **a**, **b** The mRNA and protein expression of IGF2BP3 in human nasopharyngeal epithelial cell line and 5 NPC cell lines. **c**, **d** The expression of IGF2BP3 in NPC cells after transfection with siNC and siIGF2BP3. **e**–**i** CCK8 (**e**), colony formation assays (**f**), EDU assays (**g**), transwell assays (**h**), and wound healing assays (**i**) of IGF2BP3 knockdown on cell proliferation, migration and invasion. Data was shown as the mean ± standard deviation of three independent experiments. **P < 0.01.

We determined whether IGF2BP3 had vital influences on NPC cell proliferation and metastasis through biological experiments. The results of qRT‐PCR and Western blot analyses showed a higher expression level of IGF2BP3 in five NPC cell lines than that in the normal nasopharyngeal cell NP69 (Fig. 2a, b). As such, CNE2 and 5–8 F cell lines were selected to conduct subsequent functional tests. As shown in Fig. 2c and d, siRNA against IGF2BP3 effectively reduced the endogenous expression of IGF2BP3 in NPC cells. Cell Counting Kit-8 (CCK-8), colony formation, and EDU assays were conducted to verify the biological effects of IGF2BP3 on cell proliferation. CCK8 and colony formation assays showed that IGF2BP3 knockdown significantly suppressed the viability of NPC cells (Fig. 2e, f). The EDU assays showed that the number of cells in the proliferation phase of siIGF2BP3-transfected cells was obviously reduced compared with that in the control group (Fig. 2g). Transwell assays and Wound healing assays were employed to detect NPC invasion and migration abilities under different treatments. The inhibition effect in siIGF2BP3 group in the Transwell assay indicated that knockdown of IGF2BP3 attenuated the invasive capacities of NPC cells (Fig. 2h). Moreover, the wound healing analyses demonstrated that NPC cells transfected with siIGF2BP3 showed reduced migration ability (Fig. 2i).

### Expression of IGF2BP3 is regulated by MYC

**Fig. 3:**
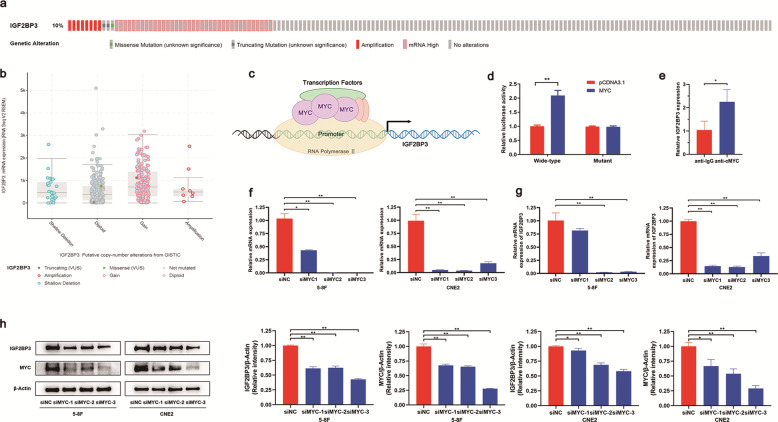
The expression of IGF2BP3 is regulated by MYC, which acts as a transcription factor. **a**, **b** The genetic alterations of IGF2BP3 in TCGA data predicted by cBioPortal. **c** Diagram of transcription factors MYC binds to the promoter region of IGF2BP3. **d** Luciferase reporter assay verified the direct target binding of MYC and IGF2BP3. **e** CHIP assay was performed to measure the connection between MYC and IGF2BP3 in NPC cells. The enrichment of ChIP is normalized by input DNA via qRT-PCR. **f**–**h** qRT-PCR and western blot results of MYC and IGF2BP3 mRNA and protein expression in 5–8 F and CNE2 cells after MYC knockdown. Data was shown as the mean ± standard deviation of three independent experiments. *P < 0.05, **P < 0.01.

To explore the precise molecular mechanism of upregulating IGF2BP3 expression in NPC tissues, we predicted genomic changes online. cBioPortal was used to analyze the TCGA data containing 496 samples. The genetic alteration of IGF2BP3 in head and neck tumors was mainly seen in high mRNA levels, indicating that IGF2BP3 was probably regulated at the transcriptional or post-transcriptional level (Fig. 3a, b). Therefore, we established the hypothesis that the increased expression of IGF2BP3 in NPC is due to the binding of related transcription factors to the promoter region of IGF2BP3 and the activation of mRNA amplification (Fig. 3c). The online websites Jaspar (http://jaspar.genereg.net/) and Genecard (https://www.genecards.org/) were employed to predict the possible transcription factors of IGF2BP3. We selected MYC, which tends to be highly expressed in most rapidly proliferating cells, for more in-depth research. In the dual luciferase reporter assays, the reporter gene vector inserted into the promoter region of IGF2BP3 showed higher luciferase activity after the overexpression of the transcription factor MYC (Fig. 3d). We also carried out a CHIP assay to examine whether MYC can directly bind to IGF2BP3, and our conjecture was confirmed (Fig. 3e). In the following qRT-PCR and WB analyses, knocking down MYC in NPC cell lines significantly reduced the expression of IGF2BP3 at the mRNA and protein levels (Fig. 3f–h). Hence, we identified that MYC binds to IGF2BP3 to upregulate its expression in NPC cells.

### IGF2BP3 affects KPNA2 mRNA stability in an m6A-dependent manner

**Fig. 4:**
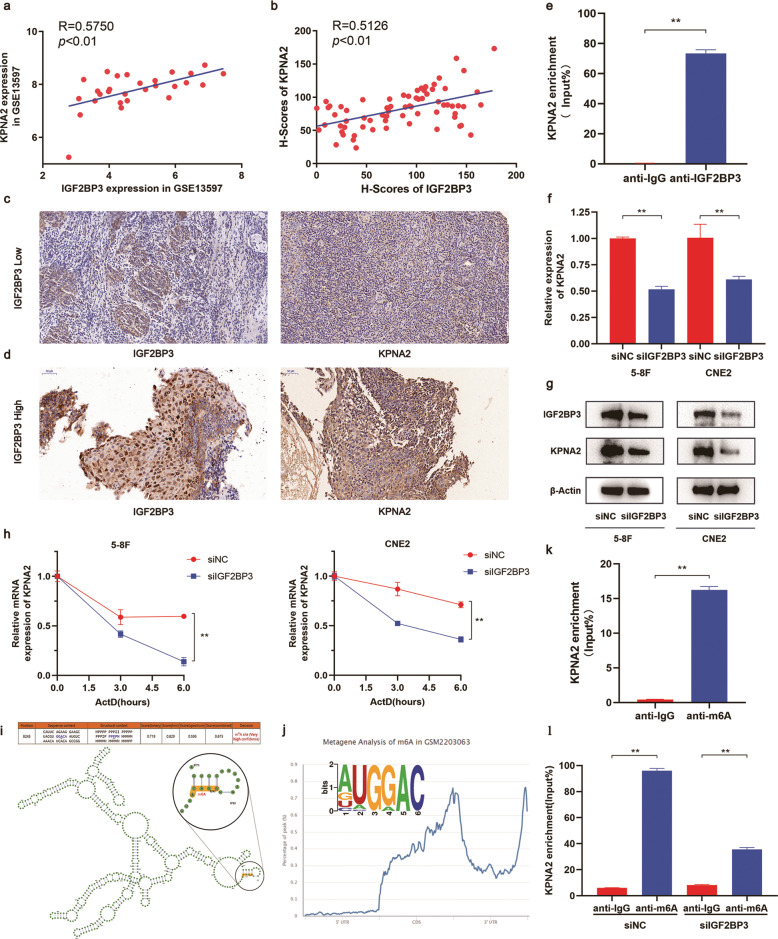
IGF2BP3 regulates the mRNA stability of KPNA2 by reading its m6A modification site. **a** Correlation between IGF2BP3 and KPNA2 in GSE13597. **b** Correlation of H-score between IGF2BP3 and KPNA2 in immunohistochemistry. **c**, **d** IHC staining of IGF2BP3 and KPNA2 in IGF2BP3 low specimens (**c**) and IGF2BP3 high specimens (**d**). **e** RIP assay analysed KPNA2 enrichment in IGF2BP3 in NPC cells. The enrichment of RIP was normalized by input RNA via qRT-PCR. **f**, **g** qRT-PCR and western blot results of IGF2BP3 and KPNA2 mRNA and protein expression in 5–8 F and CNE2 cells after IGF2BP3 knockdown. **h** Treated transfected NPC cells with actinomycin D for indicated times, and the mRNA levels of KPNA2 were measured by qRT‐PCR. **i** Possible sequence and structure of m6A sites on KPNA2 transcripts predicted by SRAMP. **j** Motif display and metagene analysis of m6A sites on KPNA2 transcripts via RMBase V2.0. **k** MeRIP-qPCR analysis of KPNA2 mRNA. **l** MeRIP-qPCR analysis of KPNA2 mRNA after IGF2BP3 knockdown, compared to the negative control. Data was shown as the mean ± standard deviation of three independent experiments. **P < 0.01.

Given that IGF2BP3 has the characteristics of RNA binding protein, we took the intersection of starBase and GEO data (GSE13597) to predict genes that might be targeted. KPNA2, a confirmed tumor promoter in a variety of cancers, gained our attention. According to the prediction results, the expression levels of IGF2BP3 and KPNA2 were positively correlated in NPC tissues (Fig. 4a–d). RIP assay was conducted to verify the direct binding between IGF2BP3 and KPNA2. The amount of KPNA2 mRNA bound by the IGF2BP3 specific antibody was significantly higher than that in the negative control IgG antibody (Fig. 4e). IGF2BP3 can mediate the RNA stability of target genes and affect the progression of different tumors. We conducted qRT-PCR and Western blot assay to explore the regulatory effects of IGF2BP3 on KPNA2 expression (Fig. 4f, g). Hence, downregulation of IGF2BP3 reduced KPNA2 expression at mRNA and protein levels. Moreover, the mRNA stability assays confirmed that IGF2BP3 knockdown suppressed the stability of KPNA2 mRNA (Fig. 4h). Hence, we provided a new insight that IGF2BP3 can bind to KPNA2 and govern its expression at post-transcriptional levels.

An online sequence-based m6A modification site predictor SRAMP (http://www.cuilab.cn/sramp) proved that the mRNA of KPNA2 exhibited high m6A methylation modifications (Fig. 4i). Meanwhile, RMBase V2.0 [16] predicted that m6A sites abound near the 3ʹUTR region of KPNA2 (Fig. 4j). We thus used MeRIP-qPCR assay. KPNA2 mRNA was enriched in m6A modification compared with the negative control (Fig. 4k). We knocked down IGF2BP3 expression in Me-RIP assays given that silencing IGF2BP3 significantly decreased KPNA2 mRNA enrichment (Fig. 4l). In summary, our data illustrated that m6A “reader” IGF2BP3 impacts KPNA2 mRNA stability in an m6A-dependent manner.

### IGF2BP3 regulates biological functions of NPC cells through KPNA2

**Fig. 5:**
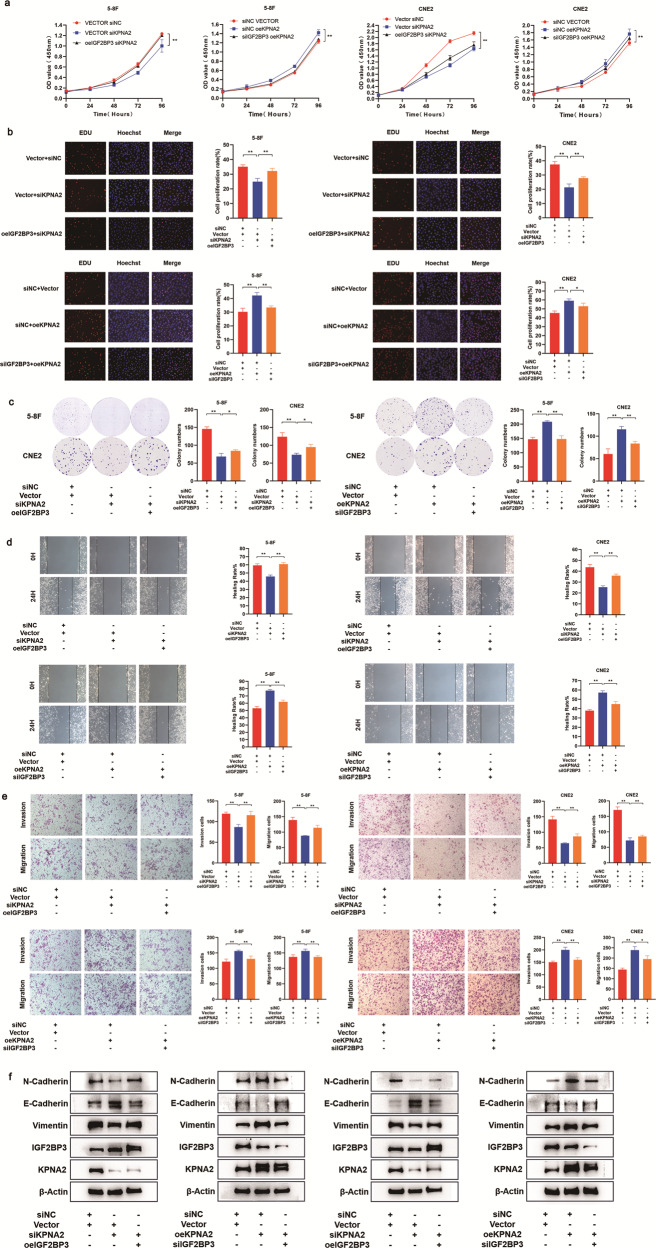
IGF2BP3 regulates proliferation and metastasis of NPC cells through KPNA2 in vitro. **a**–**g** CCK8 (**a**), EDU assays (**b**), colony formation assays (**c**), wound healing assays (**d**), and transwell assays (**e**) of IGF2BP3 regulating KPNA2 on cell proliferation and metastasis. **f** Western blot results of the knockdown or overexpression of KPNA2 and IGF2BP3. Data was shown as the mean ± standard deviation of three independent experiments. *P < 0.05, **P < 0.01.

Rescue experiments were conducted to better explore whether IGF2BP3 facilitated cell proliferation, invasion, and migration. Based on the data of CCK8 assays, knocking down KPNA2 significantly reduced cell viability, while overexpressing IGF2BP3 partly recovered these native effects in the co-transfected NPC cells (Fig. 5a). These results also could be drawn from EDU and colony formation analyses (Fig. 5b, c). IGF2BP3 inhibition attenuated the promotion of cell proliferation due to KPNA2 restoration. Furthermore, cell migration and invasion experiments described that upregulated IGF2BP3 neutralized KPNA2 knockdown-mediated suppression of cell invasion and migration, while IGF2BP3 silencing inhibited the migratory and invasive capacities that enhanced by KNPA2 overexpression (Fig. 5d, e). Additionally, Western blot assay displayed that KPNA2 knockdown significantly increased E-cadherin protein expression and decreased Vimentin and N-cadherin protein expression, and these effects could be abolished by overexpression of IGF2BP3 (Fig. 5f). All these data suggested that IGF2BP3 promotes the tumor development of NPC cells by regulating KPNA2 expressions.

### IGF2BP3 knockdown reduces the metastasis of NPC cells in vivo

**Fig. 6:**
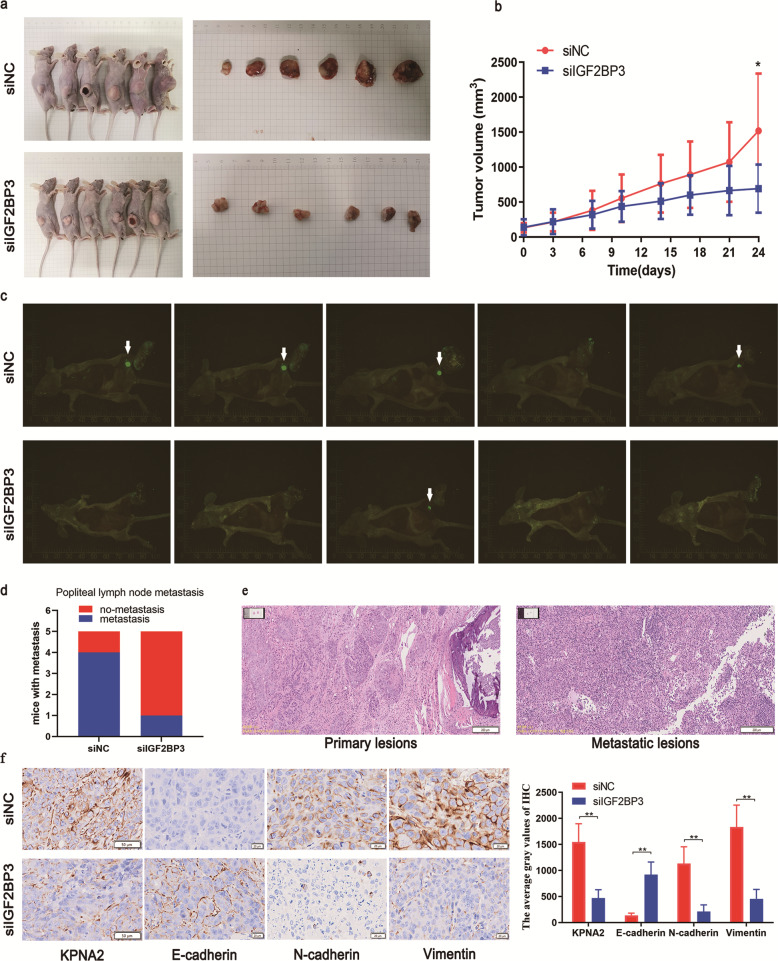
IGF2BP3 suppressed proliferation and metastasis in vivo. **a** Tumors formed by subcutaneous injection of 5–8 F cells transfected with siNC or siIGF2BP3 into nude mice. **b** The growth curves of tumor volumes were depicted in the siNC and siIGF2BP3 groups. **c** Metastatic popliteal lymph nodes were visualized by fluorescent staining. **d** The incidence of popliteal lymph node metastasis of each group. **e** HE staining of popliteal metastasis. **f** The expression of KPNA2, E-cadherin, N-cadherin and Vimentin in tumors assessed by IHC. Data was shown as the mean ± standard deviation of three independent experiments. *P < 0.05, **P < 0.01.

To further ascertain the effects of IGF2BP3 on the tumorigenesis of NPC cells in vivo, we successfully built nude mouse models. As illustrated in the subcutaneous tumor models, the tumor volumes and weights in IGF2BP3 silenced groups were lower than those in the control groups (Fig. 6a, b). Moreover, based on the data obtained from the established axillary lymph node metastasis models, we found that silencing IGF2BP3 decreased the number of mice with lymph node metastases compared with the control (Fig. 6c, d). The metastatic foci were identified using HE staining (Fig. 6e). In addition, IHC methods were employed to detect the expression of KPNA2 and important markers of EMT in tumor tissues of mice, including E-cadherin, N-cadherin, and Vimentin. As predicted, the E-cadherin expression increased in siIGF2BP3 groups, whereas the expression levels of KPNA2, N-cadherin, and Vimentin were subsided (Fig. 6f). All the above results confirmed that silencing IGF2BP3 represses cell growth and metastasis in vivo.

## Discussion

Accumulating evidence on RNA modification has been gradually revealed in recent decades, among which m6A modification, is considered the most ubiquitous reversible RNA post-transcriptional modification in eukaryotes. Without the alterations in base pairing, m6A modification exerted a decisive influence on the fate of mRNAs and non-coding RNAs. As such, the biological processes of RNA, including decay, splicing, translation, and transport, were affected to varying degrees, leading to an imbalance in the body’s immune infiltration, glycolytic metabolism, stem cell differentiation, and tumorigenesis [17–20]. In recent years, scholars reported that m6A regulatory protein promotes the malignant progression of NPC. As an example of such mechanism, METTL3 mediates m6A modification of TRIM11 mRNA to stabilize its transcript via the m6A reader IGF2BP2-dependent pathway, thereby exacerbating drug resistance in NPC [21]. Meanwhile, the bioinformatic analysis demonstrated that m6A-related genes were dysregulated in NPC, and this dysregulation was significantly correlated with poor prognosis [22]. In the present study, we were inspired by the systematic research of Li et al.; they reported that IGF2BP1/2/3, YTHDF1/3, HNRNPA2B1, and VIRMA exhibited copy number variations in amplification across 33 cancer types. Given that we conducted a preliminary study on the function of IGF2BP3 as a tumor promoter in esophageal cancer [23], we aimed to elucidate the expression characteristics and biological functions of IGF2BP3 in NPC. This work provides a basis for developing a new method of tumor treatment from an m6A perspective.

An in-depth study of m6A regulators showed that IGF2BP3 is tightly associated with the occurrence and development of pancreatic cancer. For example, Zhang et al. found that IGF2BP3 is generally up-regulated in 18 TCGA tumors, and the high expression level of IGF2BP3 affects the immune microenvironment and drug sensitivity, leading to poor prognosis [24]. Simultaneously, IGF2BP3 was identified as an independent prognostic indicator in renal papillary cell carcinoma [25], hematological malignancies [26], and prostate cancer [27]. Consistent with these studies, we found that IGF2BP3 was upregulated in patients with NPC and NPC cells. Moreover, statistics showed that the expression of IGF2BP3 was significantly related to pathological N stage and TNM stage in 70 patients. The higher the expression of IGF2BP3, the poorer the prognosis of the patient will be. Univariate and multivariate analyses concluded that IGF2BP3 is an independent risk factor for NPC. Functional experiments presented the suppressive function of IGF2BP3 knockdown on cell proliferation, invasion, and metastasis of NPC in vitro and in vivo. These results implied the pivotal role of IGF2BP3 in NPC tumor development. In this regard, the mechanism of IGF2BP3 in NPC should be further explored.

Multiple putative mechanisms have been revealed for the aberrantly high expression of IGF2BP3. For instance, Huang et al. indicated that IGF2BP3 could be protected by USP11 from degradation via deubiquitylation [28]. Previous work by Zhou et al. elucidated that the genomic alteration of IGF2BP3 in gastric cancer is mainly caused by mRNA upregulation, and it is regulated by miR-34a [29]. A similar mechanism applies to miR-125a-5p, which binds directly to the 3ʹUTR of IGF2BP3 mRNA, causing mRNA amplification. In the present study, we used cBioPortal and found that the major genetic alterations in IGF2BP3 in HNSCC lies in amplification at the mRNA level. This finding indicates the importance of transcriptional or post-transcriptional regulation. MYC acts as a transcriptional regulator that directly binds to the promoter region of IGF2BP3. qRT-PCR and WB assays confirmed that MYC regulates IGF2BP3. Luciferase reporter and CHIP assays indicated that MYC binds to the IGF2BP3 promoter region as a transcriptional factor to govern NPC progression. Surprisingly, some scholars have suggested that IGF2BPs protect MYC mRNA from decay in m6A manner [30, 31]. MYC is thus considered a target gene of IGF2BP3. In most human tumors, amplified MYC tends to regulate cell growth proliferation, self-renewal, and pluripotency as well as DNA replication [32]. In NPC, MYC directly or indirectly regulates genes, such as miR-141 [33] and miR-200c, [34] for the general cellular response. Based on our research, an interesting phenomenon appeared, wherein a regulatory loop was formed between IGF2BP3 and MYC. This phenomenon may trigger a cascade and magnify the effect of NPC and constitute the essential hub of a regulatory network for controlling fundamental cellular processes. The present study provides new insights into the possible mechanism of IGF2BP3 overexpression from the perspective of the promoter. Other mechanisms that initiate the amplification of IGF2BP3 mRNA levels, such as some related miRNAs, are being explored.

IGF2BP3 is a member of the IGF2BP family. Previous studies focused on the mechanism of IGF2BP3 as an RNA-binding protein that dictates RNA life cycle. Nonetheless, as an m6A reader, IGF2BP3 plays an irreplaceable role in different types of malignancies. Highly expressed IGF2BP3 can directly bind to the m6A-modified region of target mRNA, thereby promoting the stability and expression of specific genes, such as TMBIM6 [35], VEGF [36], and ABCB1 [37], leading to tumor progression, angiogenesis, and chemoresistance. In the present study, we used starBase and GEO data to predict genes where IGF2BP3 may bind. KPNA2 not only binds to IGF2BP3 but its expression is positively correlated with the latter. qRT-PCR and WB results validated that silencing IGF2BP3 represses KPNA2 RNA and protein expression. Subsequently, RNA modification online database RMBase V2.0 predicts the presence of m6A sites in KPNA2 3ʹUTR. MeRIP assay was then performed to verify the enrichment of m6A modification in KPNA2 sequence. RIP and MeRIP assay further proved that IGF2BP3 effectively binds to KPNA2, and silencing IGF2BP3 significantly suppresses the m6A methylation level of KPNA2. Furthermore, IGF2BP3 promotes KPNA2 mRNA stability in NPC cells treated with ActD. Final rescue assays were conducted to verify the ability of KPNA2 to offset the IGF2BP3 overexpression‐mediated function on the progression of NPC. Hence, IGF2BP3 could be an m6A-modification regulator of KPNA2 in NPC.

The novelty of our study is that we propose an essential regulatory loop between MYC and IGF2BP3 in NPC. IGF2BP3 played a critical role in NPC progression by affecting the mRNA stability of m6A-modified KPNA2. However, the present study did not mention the mechanism through which KPNA2 obtains the m6A modification site in NPC, which is a major drawback. Future work will be dedicated to identifying the possible m6A writer of KPNA2. In conclusion, MYC-activated IGF2BP3 promoted the proliferation and metastasis of NPC by enhancing the mRNA stability of KPNA2. Hence, IGF2BP3 may act as a new biomarker in NPC treatment.

## Materials and methods

### Clinical specimens

Seventy frozen NPC tissues and ten normal nasopharyngeal epithelium tissues were obtained from Jiangsu Cancer Hospital (Nanjing, China) between February 2006 and December 2012. These patients did not receive chemotherapy or radiotherapy before surgery. Moreover, all tissue samples were confirmed by pathologists. Institutional Ethical Review Board of Jiangsu Cancer Hospital approved this research, and each patient provided written informed consent.

### Cell culture

NPC cell lines, namely, CNE1, CNE2, SUNE1, 5–8 F, and 6-10B, were maintained in (RPMI)1640 medium (Corning, USA) supplemented with 10% fetal bovine serum (Gibco) in a humidified 5% CO_2_ incubator at 37 °C. The cells were provided by Jiangsu Cancer Institute and authenticated by STR.

### Cell transfection

Small interfering RNAs (siRNAs) of MYC, KPNA2, and IGF2BP3 were designed and synthesized by RiboBio Company (Guangzhou, China). Plasmids IGF2BP3 and KPNA2 were purchased from Vigenebio Biosciences Company (Jinan, China). According to the manufacturer’s instructions, the siRNAs and plasmid were transfected into NPC cells by using Lipofectamine 2000 (Invitrogen, USA).

### Wound healing assay

Cells (5 × 10^5^ cells/well) were seeded into a 6-well plate and incubated in a serum-free medium for 48 h. A 200 μL pipette tip was used to gently create a linear wound. Migratory cells were observed at ×100 magnification under the light microscope at two-time points of 0 and 24 h.

### Transwell assay

The transfected cells were resuspended (3 × 10^4^ cells per well) into 200 μL of serum-free medium and plated in the upper chamber. The lower chambers were filled with 20% FBS and 500 μL of RPMI-1640. After 36 h of incubation, the upper chamber was fixed and stained. An inverted microscope was used to count the invading cells.

### In vivo nude mouse xenograft tumor models

Twenty-two 6–8 weeks old BALB/c nude mice were acquired from Yangzhou University Medical Center (Yangzhou, China), and were randomly divided into siIGF2BP3 and siNC control groups. In vivo siIGF2BP and control siRNA expressing green fluorescent protein (GFP) were synthesized by RiboBio (Guangzhou, China). Intratumorally injected these mice (5nmol siRNA/20 g) every 3 days until the mice were euthanized. The tumors from different groups were weighed. GFP was used to observe popliteal lymph node metastasis in mice. Then, popliteal lymph nodes were enucleated. Sections of the lymph nodes were stained with hematoxylin and eosin (H&E) for pathology verification. The expression of KPNA2, E-cadherin, N-cadherin and Vimentin in the metastatic lymph nodes was determined by immunohistochemical (IHC) analysis. All animal experiments were authorized by the Animal Science Committee of the Animal Science of Nanjing, China.

### Cell counting kit-8 assay

A CCK-8 kit (Dojindo, Japan) to measure cell proliferation. A total of 3000 cells per well were cultured in five replicate wells for each experimental group in 96-well plates after transfection. Each well was added with 10 μL of Cell Counting Kit-8 (CCK-8) reagent and 100 μL of RPMI-1640 medium. Cell proliferation and viability were detected by recording the absorbance at 450 nm daily for 4 consecutive days.

### Colony formation assay

In brief, 1.5 × 10^3^ treated cells were seeded in six-well plates with three repetitions. After 14 days of cultivation, the colonies were washed with PBS, fixed by paraformaldehyde (Sigma), and stained with 0.1% crystal violet solution for further analysis.

### EDU

Cells were cultured into 96-well plates with a density of 10 × 10^5^ cells per well and treated with 100 μL of the medium containing 50 μM EdU at 37 °C for 2 h. The cells were then fixed with 4% formaldehyde for 0.5 h and permeabilized with 0.1% Triton X-100 for 20 min. The cells were stained with Apollo Fluorescent Dye according to the instruction of the EdU cell proliferation detection (imaging detection) kit (RiboBio, China). Finally, the cells were observed under a fluorescence microscope.

### Quantitative real-time PCR

According to the manufacturer’s manuals, total cellular RNAs were extracted by TRIzol reagent (Invitrogen). Complementary DNA (cDNA) was synthesized using the Prime Script™ RT reagent kit (TAKARA, Shiga, Japan). The relative expression levels of MYC, IGF2BP3, and KPNA2 in the cells were compared with that of GAPDH. Expression fold changes were computed using 2−ΔΔCt method. The following primers were used: GAPDH, forward primer 5ʹ-AAGGCTGTGGGCAAGG-3ʹ and reverse primer 5ʹ-TGGAGGAGTGGGTGTCG-3ʹ; MYC, forward primer 5ʹ-TGCTCCATGAGGAGACACC-3ʹ and reverse primer 5ʹ-CTTTTCCACAGAAACAACATCG-3ʹ; IGF2BP3, forward primer 5ʹ-CCATAGAAGTTGAGCACTCGGTCC-3ʹ and reverse primer 5ʹ-TCTCCACCACTCCATACTGGACTAG-3ʹ; and KPNA2, forward primer 5ʹ-GTGGGCCGTGACCAACTATAC-3ʹ and reverse primer 5ʹ-CATCAACGGTTCTATTATGCCAC-3ʹ.

### Western blot analysis

Total protein was extracted from NPC cell lines by using RIPA buffer, a phosphatase inhibitor, and PMSF (Beyotime). Protein concentration was detected with a NanoDrop 2000 spectrophotometer (Thermo Fisher Scientific). An equal amount of the protein sample was separated via sodium dodecyl sulfate–polyacrylamide gel electrophoresis and then transferred onto polyvinylidene fluoride membranes (Millipore, USA). The membranes were incubated with different primary antibodies including β-actin (1:1000, Cell Signaling Technology, USA), MYC (1:1000, Cell Signaling Technology, USA), IGF2BP3 (1/1000, Abcam, USA), KPNA2 (1:1000, Cell Signaling Technology, USA), N-cadherin (1:1000, Cell Signaling Technology, USA), E-cadherin (1:1000, Cell Signaling Technology, USA), vimentin (1:1000, Cell Signaling Technology, USA), and goat anti-rabbit IgG (1:40000, Abbkine, USA). Immunoreactive bands were visualized with an ECL detection reagent (Millipore, MA, USA).

### ChIP assay

ChIP assay was performed with a chromatin immunoprecipitation kit (Millipore, USA) following the manufacturer’s instruction. Anti-IGF2BP3 antibody was obtained from Abcam. In brief, a final concentration of 1% formaldehyde was used to fix chromatin from 10 million NPC cells from different treatment groups and sonicated to about 200–1000 bp fragments. Antibody-bound protein A/G Dynabeads were incubated with chromatin under sonication at 4 °C overnight. After four rounds of washing, the precipitated protein–DNA complex was reverse-crosslinked at 62 °C for 2 h and 95 °C for 10 min. DNA was purified using phenol-chloroform. The CHIP samples were analyzed via qRT-PCR. The primer sequences of MYC-IGF2BP3 were as follow: forward primer 5ʹ-CTCTCCCAATCTCGTTTCCCC-3ʹ; reverse primer 5ʹ-AAAATCCGCTCCGAGTGTCC-3ʹ.

### Dual-luciferase reporter assays

293 T cells were plated at 1.5 × 10^4^ cells/well in 96-well plates overnight. The cells were co-transfected with WT-IGF2BP3 or MUT-IGF2BP3 reporter plasmid and MYC OE or Control. After 48 hours of incubation, relative luciferase activity was determined by Dual-Luciferase Reporter Assay System (Promega) according to the manufacturer’s protocol.

### RIP assay

RIP assay was performed with Magna RIP Kit (Billerica, MA, USA). NPC cells were lysed in complete RIP lysis buffer containing magnetic beads conjugated with anti-m6A (Synaptic Systems, German), anti-IGF2BP3, or control anti-IgG antibody at 4 °C overnight. The protein was digested by incubation with proteinase K after the magnetic beads were washed. The purified RNA was used for qRT-PCR analysis.

### IHC

The sections were incubated with primary antibody at 4 °C overnight. A kit (ZSGB BIO Inc.) was used to immunostain the DAB chromogen, followed by nuclear staining with hematoxylin.

### Statistical analysis

All statistical analyses were carried out using SPSS 20.0 software and GraphPad Prism 8.0 software. Data were presented as mean ± SD. Unpaired Student’s *t* test was applied for a two-group comparison of the other assays. Chi-square test was used to explore differences among categorical variables. Survival analysis was performed using Kaplan–Meier method with log-rank test. Significance was considered at *P* < 0.05 (*P < 0.05, **P < 0.01).

## Supplementary information

Supply Figure 1

Supply Figure 2

## Data Availability

The data that support the findings of this study are available from the corresponding author upon reasonable request.
