# Supplementary figures and images for: MYC-activated RNA N6-methyladenosine reader IGF2BP3 promotes cell proliferation and metastasis in nasopharyngeal carcinoma
Source: Cell Death Discov. 2022 Feb 8;8:53. doi: 10.1038/s41420-022-00844-6 (PMC8826370; doi:10.1038/s41420-022-00844-6)

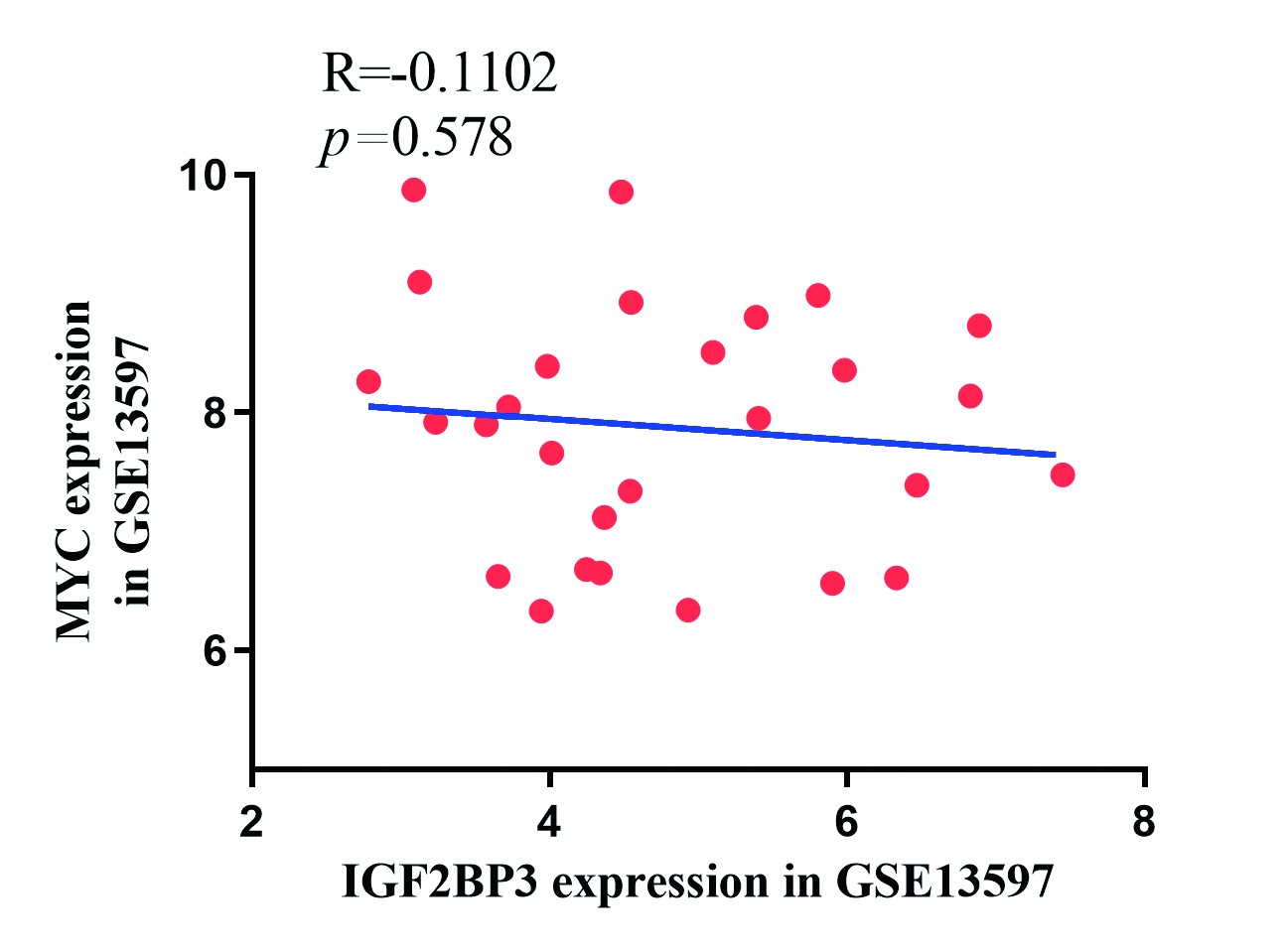

Supplement: Supplementary file 1 — Supply Figure 1 [file 41420_2022_844_MOESM1_ESM.tif]

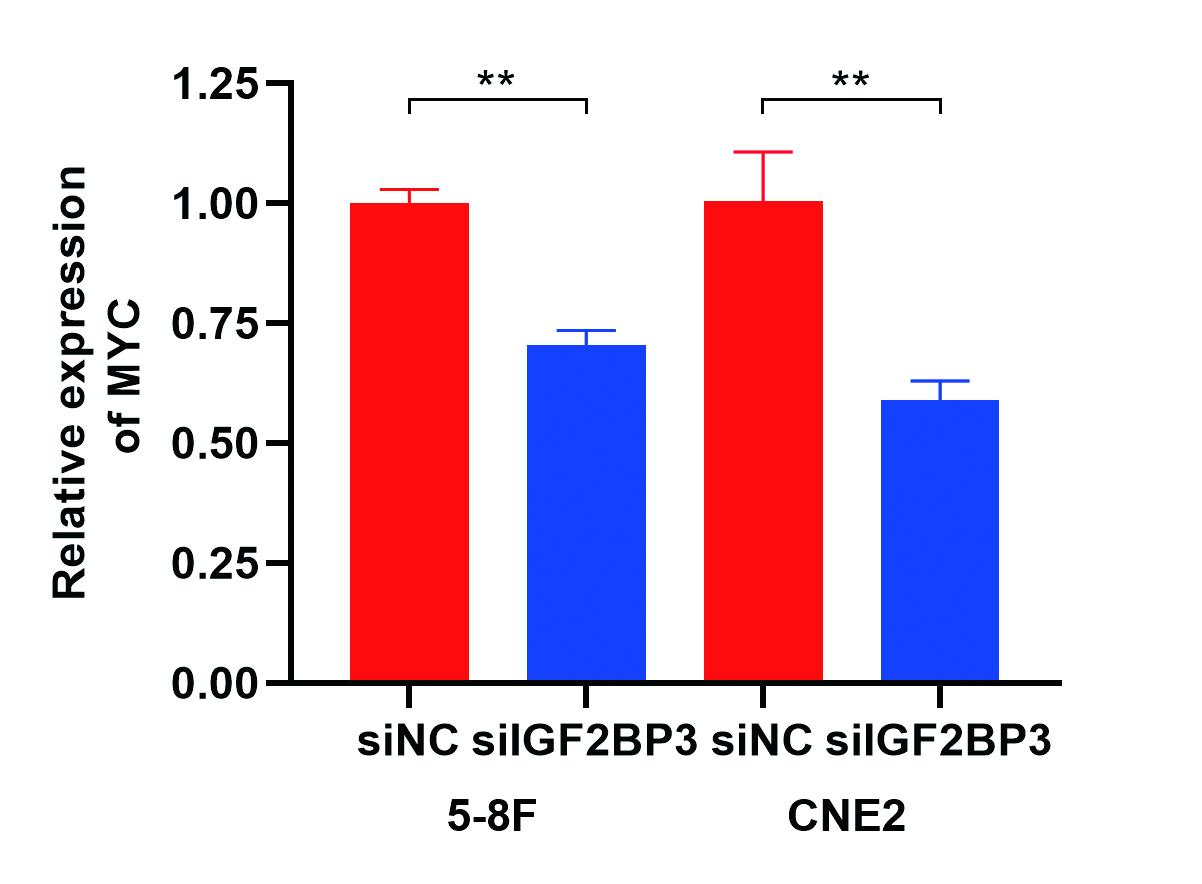

Supplement: Supplementary file 2 — Supply Figure 2 [file 41420_2022_844_MOESM2_ESM.tif]
